# Brazilian Consensus on Psoriasis 2020 and Treatment Algorithm of the Brazilian Society of Dermatology^☆^^☆☆^

**DOI:** 10.1016/j.abd.2021.03.007

**Published:** 2021-09-23

**Authors:** Ricardo Romiti, André Vicente E. de Carvalho, Gleison V. Duarte, Grupo de Trabalho do Consenso Brasileiro de Psoríase da Sociedade Brasileira de Dermatologia

**Affiliations:** a Dermatology Department of Hospital das Clínicas da Universidade de São Paulo, SP, Brazil; b Hospital Moinhos de Vento de Porto Alegre, Porto Alegre, RS, Brazil; c Instituto Bahiano de Imunoterapias, Salvador, BA, Brazil

Dear Editor,

The elucidation of pathophysiological mechanisms and the development of new treatments for psoriasis require periodic updates in the publication of consensuses, algorithms and treatment guides.

In Brazil, the ethnic composition, increased longevity, in addition to climatic and insolation characteristics may imply unique epidemiological data and different regional prevalence rates of psoriasis, in addition to influence disease severity and therapeutic response. Recent data from the Brazilian Society of Dermatology estimate the prevalence of psoriasis in Brazil at 1.31%, with 1.15% (95% CI 0.90% to 1.43%) in women and 1.47% (95% CI 1.11% to 1.82%) in men (p = 0.22). An increase in the prevalence of psoriasis (p < 0.01) was identified in relation to age groups, which was 0.58% (95% CI 0.31% to 0.84%) under the age of 30; 1.39% (95% CI 1.10% to 1.74%) between 30 and 60 years old; and 2.29% (95% CI 1.71% to 2.84%) in individuals over 60 years. The geographical regions of the country differed in terms of disease prevalence (p = 0.02), with higher indicators in the South and Southeast regions, in contrast to the Midwest, North and Northeast regions.^1^

In parallel, 73.4% of Brazilian patients with moderate to severe psoriasis report impaired health-related quality of life.^2^

The Brazilian Consensus on Psoriasis 2020 and the Treatment Algorithm of the Brazilian Society of Dermatology (SBD), created with the collaboration of experts from all regions of Brazil is shown below (Fig. 1).

**Figure 1:**
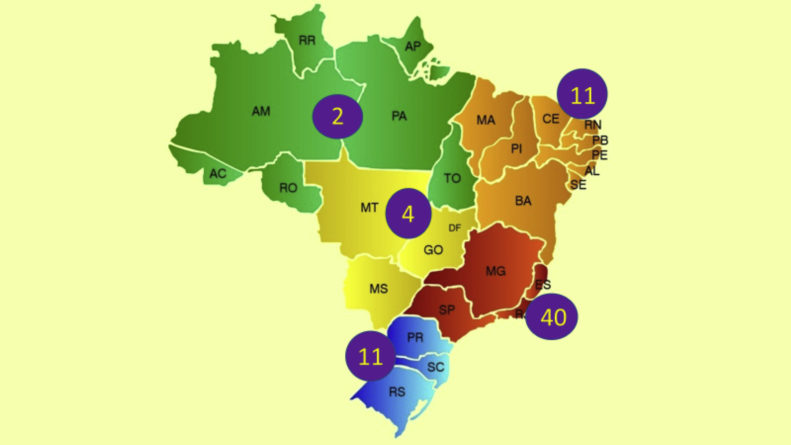
Geographical distribution of Brazilian experts participating in the Brazilian Consensus on Psoriasis 2020.

In preparing this consensus, the stratification of the levels of evidence and their grade of recommendation was used, according to the project guidelines of the Brazilian Medical Association (AMB), described below:

Grade of recommendation and strength of evidence^3^

A: Experimental or observational studies of higher consistency.

B: Experimental or observational studies of lower consistency.

C: Case reports (uncontrolled studies).

D: Opinion without critical evaluation consensus based, physiological studies or animal models.

In this document, we use the Delphi tool to obtain answers to non-consensual questions in the literature, through the anonymous collection of data among specialists on the subject. The Delphi method is defined as “a data collection technique used to obtain consensus from a group of experts on a particular subject”.^4^ Thus, a new treatment flowchart for severe psoriasis was validated and strategies were defined for the migration of therapies, designed for adoption in the Brazilian public or private health context, based on hearing the opinions of experts engaged in these health systems (Fig. 2).

**Figure 2:**
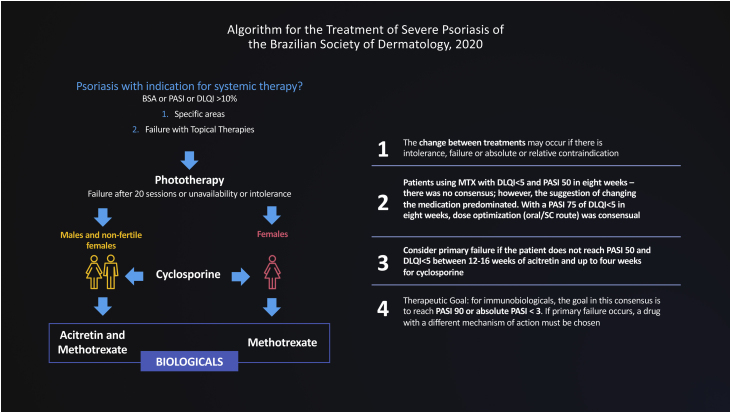
Algorithm for the Treatment of Severe Psoriasis of the Brazilian Society of Dermatology, 2020.

Two rounds were held with the participation of 66 dermatologists who authored this consensus, from all regions of the country, with experience in the treatment of psoriasis. The online voting system (Survey Monkey®) was used, and the agreement of at least 70% of the experts was considered a consensus. The data obtained were statistically analyzed. The sample was obtained by appointment and submitted to a randomness test, which resulted in the non-rejection of randomness, with a significance of 5%. The results are shown in Table 1.

**Table 1:** Classification of respondents' opinions, per question, Brazil – 2020. Source: Study data.

Classification of respondents' opinions, per question, Brazil – 2020		
Consensus criterion: 70% of agreement or more.		
Questions	Answers with the highest percentage of agreement	Classification
Q1: What is the criterion for changing from one therapy to another?	Unavailability, intolerance, failure or absolute or relative contraindication	Consensus
Q2: What to do if the patient does not reach PASI 50 and DLQI < 5 (primary failure) with oral methotrexate at a dose of 15 mg/week within 8 weeks, with full access/availability?	1. Medication change: 54%	Dissensus
	2 MTX dose optimization up to 25 mg/week, regardless of administration route: 46%	
Q3: If the patient does not reach PASI 75 and DLQI < 5 (secondary failure) with oral methotrexate at a dose of 15 mg/week within 8 weeks, what is the suggested approach when there is full access/availability?	MTX dose optimization up to 25 mg/week, regardless of administration route (oral/parenteral)	Consensus
Q4: If the patient does not reach PASI 50 and DLQI < 5, acitretin failure should be considered after:	12–16 weeks	Consensus
Q5: If the patient does not reach PASI 50 and DLQI < 5, primary failure of cyclosporine (up to 5 mg/kg) should be considered in:	Up to 4 weeks	Consensus
Q6: What is the therapeutic goal to be achieved in patients using biologicals?	PASI 90 or absolute PASI <3	Consensus
Q7: What is your degree of agreement with the sentence: "In patients with primary failure with an immunobiological, the medication should be changed to another one with a different mechanism of action"?	70% of agreement	Consensus
Q8: What is your degree of agreement with the sentence: "Patients with secondary failure with a biological mechanism of action can benefit from a change to a drug of the same class"?	51% of agreement	Dissensus
Q9: From 1–10 what is your degree of agreement with the proposed flowchart? (1 = strongly disagree; 10 = strongly agree).	86.4% of agreement	Consensus

Mediated by the Brazilian Society of Dermatology, this instrument represents progress in the standardization of conduct, whether in public or private care, based on what we have available or aspire for the treatment of severe psoriasis. The existence of dissensus stimulates further debate on controversial and unanswered topics in the medical literature.

The high percentage of agreement in the other topics provides subsidies to professionals working in the area for the best therapeutic choices, instead of decisions based solely on the prescriber's experience. Such transparency is essential for everyone involved, whether managers of the supplementary health system or the Brazilian Unified Health System (SUS), physicians, patients, their families, and patient associations.^5^

## Financial support

Brazilian Society of Dermatology (SBD).

## Authors' contributions

Ricardo Romiti: Design and planning of the study; data collection, or data analysis and interpretation; statistical analysis; drafting and editing of the manuscript or critical review of important intellectual content; collection, analysis, and interpretation of data; effective participation in research orientation; critical review of the literature; approval of the final version of the manuscript.

André Vicente Esteves de Carvalho: Design and planning of the study; data collection, or data analysis and interpretation; statistical analysis; drafting and editing of the manuscript or critical review of important intellectual content; collection, analysis, and interpretation of data; effective participation in research orientation; critical review of the literature; approval of the final version of the manuscript.

Gleison V Duarte: Design and planning of the study; data collection, or data analysis and interpretation; statistical analysis; drafting and editing of the manuscript or critical review of important intellectual content; collection, analysis, and interpretation of data; effective participation in research orientation; critical review of the literature; approval of the final version of the manuscript.

## Conflict of interest

Romiti R is/has served as a scientific consultant, speaker, or clinical study investigator for Abbvie, Boehringer-Ingelheim, Galderma, Janssen, Lilly, Leo-Pharma, Novartis, Pierre-Fabre, Pfizer, UCB, and TEVA.Carvalho AVE is/has served as a scientific consultant, speaker, or clinical study investigator for Abbvie, Boehringer-Ingelheim, Janssen, Lilly, Leo-Pharma, Novartis, and UCB.Duarte GV is/has served as a scientific consultant, speaker, or clinical study investigator for Abbvie, Bayer, Janssen, Leo-Pharma, Galderma, Novartis, Pfizer, and UCB.

## Figures and Tables

**Table:** 

Authors of the Brazilian Consensus on Psoriasis 2020
Silvio Alencar Marques (SP), Luciane D. B. Miot (SP), Maria Cecilia de C. Bortoletto (SP), Arnóbio da Penha Pachêco (RN), Adriane Reichert Faria (PR), Caio Cesar S. de Castro (PR), Luciena C. Martins Ortigosa (SP), Xinaida Lima (CE), Sueli Carneiro (RJ), Marcelo Pinheiro (SP), Mayra Ianhez (GO), Monica Nunes de Souza Santos (AM), Roberta Buense Bedrikow (SP), Rosana Lazzarini (SP), João Carlos R. Avelleira (RJ), Aline Bressan (RJ), André Luís da Silva Hirayama (SP), Sineida B. Ferreira (PR), Adriana M. Porro (SP), Alexandre Gripp (RJ), Ivonise Follador (BA), Juliana Nakano (SP), Andréa Machado C. Ramos (MG), Aripuanã Terena (MG), Francisca Regina O. Carneiro (PA), Vivianne Lira da C. Costa (RN), Anber A. Tanaka (PR), Lincoln Fabricio (PR), Leticia Oba Galvão (DF), Gladys A. Martins (DF), Ricardo Romiti (SP), Clarice Kobata (SP), Michelle dos Santos Diniz (MG), Cacilda da Silva Souza (SP), Renata F. Magalhães (SP), Dimitri Luz (SP), André Vicente E. de Carvalho (RS), Jaquelini Silva (RS), Claudia Maia (RJ), Paulo A. Oldani Felix (RJ), Wagner Galvão (SP), Maria Victoria Suárez Restrepo (SP), Cynthia C. F. Mota (SP), Eduardo L. Martins (SP), Daniel H. Nunes (SC), Andreia Costa (SP), Mauricio Conti (SC), Esther B. Palitot (PB), Ana Carolina B Arruda (SP), Claudio Lerer (DF), Livia Souto (RJ), Roberto Souto (RJ), Jane Bonfá (RJ), Verônica Bogado (RJ), Sidney Augusto da C. Costa (RN), Domingos Jordão Neto (SP), Luiza O. Keiko (SP), Bruna Falcone (SP), Luna Azulay Abulafia (RJ), Aldejane Gurgel de A. Rodrigues (PE), Tania F. Cestari (RS), Juliana Bozza (RS), Marcelo Arnone (SP), Maria Denise F. Takahashi (SP), Heitor de Sá Gonçalves (CE), Paulo Eduardo de Sá Gonçalves (CE), Gleison V. Duarte (BA), Maria de Fátima S. Paim de Oliveira (BA).
